# Psychische Gesundheitskompetenz: Deutschsprachige Version einer modifizierten Mental Health Literacy Scale (MHLS-DE) – Psychometrische Überprüfung und Prädiktive Faktoren

**DOI:** 10.1055/a-2694-5684

**Published:** 2025-10-21

**Authors:** Gebhard Sammer, Pia Höhler, Andreas Jung, Christoph Mulert

**Affiliations:** 1 Klinische Psychologie, Justus-Liebig-Universität Gießen; 2 EX-IN Hessen e.V. Marburg; 3 Gießener Aktionsbündnis für Seelische Gesundheit e.V., Gießen

**Keywords:** Psychische Gesundheitskompetenz, Public Health, Prädiktive Faktoren, Mental Health Literacy, Public Health, Predicitve Factors

## Abstract

Psychische Gesundheitskompetenz – Mental Health Literacy (MHL) - gilt als Schlüsselkonzept für den Umgang mit psychischen Erkrankungen. Es umfasst Wissen über Krankheiten, Risikofaktoren, Hilfesuchverhalten und Einstellungen zu psychischen Erkrankungen. Auch Scham wird mit diesen Faktoren in Verbindung gebracht. In dieser Studie wurde eine modifizierte deutsche Version (MHLS-DE) entwickelt und an einer online-Stichprobe (N=533) überprüft. Es wurden psychometrische Eigenschaften der MHLS-DE und die Rolle von Scham als Mediator für MHL untersucht. Die MHLS-DE erzielte eine gute Reliabilität (Cronbach’s α=0,846) und strukturelle Validität. Mediationseffekte durch Schamerleben zeigten sich, moduliert durch Geschlecht, für den Zusammenhang zwischen psychischer Flexibilität und MHL. Trotz weiterbestehender konzeptioneller Unklarheiten lässt sich Psychische Gesundheitskompetenz mit der MHLS-DE zuverlässig erfassen. Die Mediationsanalyse verdeutlichte die Rolle von Schamgefühlen für MHL, die bei Interventionen zur Verbesserung des MHL mit einbezogen werden sollen.

## Abstract (en)

Mental health literacy (MHL) is considered a key concept in dealing with mental illness. It encompasses knowledge about illnesses, risk factors, help-seeking behavior, and attitudes toward mental illness. Shame is also associated with these factors. In this study, a modified German version (MHLS-DE) was developed and tested on an online sample (N=533). The psychometric properties of the MHLS-DE and the role of shame as a mediator of MHL were investigated. The MHLS-DE achieved good reliability (Cronbach’s χ=0.846) and structural validity. Gender-modulated mediation effects through shame experiences were found for the relationship between psychological flexibility and MHL. Despite ongoing conceptual ambiguities, mental health literacy can be reliably assessed with the MHLS-DE. The mediation analysis highlighted the role of feelings of shame in MHL, which should be considered in interventions to improve MHL.

## Einleitung

Obwohl 2024 in Deutschland 34% der Bevölkerung ab 18 Jahren angaben, unter psychischen Beschwerden zu leiden, nahmen 24% davon keine Behandlung in Anspruch 1 2 . Als wesentlicher Faktor für diese Behandlungslücke werden Wissensdefizite zu Psychischer Gesundheit genannt 3 . Eine deutschlandweite Querschnittsbefragung zeigte, dass es vielen schwer fällt zu verstehen, wie man mit psychischen Problemen umgehen oder psychisch gesund bleiben kann 4 . Auch die Stigmatisierung psychischer Erkrankung erschwert die Inanspruchnahme von Unterstützung 5 . Dass bessere psychische Gesundheitskompetenz stigmatisierende Einstellungen gegenüber psychisch erkrankten Menschen reduziert, wird im Ergebnisbericht zur Psychischen Gesundheitskompetenz in Deutschland thematisiert 6 .

Gesundheitskompetenz, als freie Übersetzung des Begriffs „Health Literacy“ beschreibt die Herausforderung, relevante Gesundheitsinformationen zu filtern, zu bewerten und darauf basierende Entscheidungen für das eigene oder das Gesundheitsverhalten von Angehörigen zu treffen 4 . Aufbauend auf diesem Konzept entwickelte sich die „Psychische Gesundheitskompetenz“ (engl. Mental Health Literacy, MHL) als eigener Ansatz weiter. Die erste Definition zu MHL wurde im Jahr 1997 in Bezug auf schulbildungsorientierte Gesundheitserziehung von Kindern und Jugendlichen vorgeschlagen und fokussierte auf Wissen und Überzeugungen zu psychischen Störungen, die zur Erkennung, Behandlung oder Prävention beitragen 7 . Dieses wissensbasierte Konzept wurde auf Erwachsene ausgeweitet 8 9 , aber auch festgehalten, dass trotz der theoretischen und praktischen Relevanz von MHL, eine begriffliche Unschärfe sowie ein Mangel an validierten Instrumenten zur zuverlässigen Erfassung von MHL bestünde 10 .

Ein zentrales Instrument zur Erfassung von psychischer Gesundheitskompetenz ist die Mental Health Literacy Scale (MHLS) von O’Connor & Casey 11 . Mit 35 Items werden 6 Domänen (in Klammern die Anzahl von Items) „Fähigkeit, Störungen zu erkennen“ (8), „Wissen, wo man Informationen findet“ (4), „Wissen über Risikofaktoren und Ursachen“ (2), „Wissen über Selbstfürsorge“ (2), „Wissen über verfügbare professionelle Hilfe“ (3) und „Einstellungen, die das Erkennen oder angemessenes Verhalten bei der Suche nach Hilfe fördern“ (16) erfasst. Die Faktorenanalyse des Fragebogens ergab jedoch nur eine 4-Faktoren- Lösung mit niedrigen Kommunalitäten. Dieses Ergebnis lasse auch die Eindimensionalität des Konstrukts vermuten 11 . Seitdem wurden mehrere Übersetzungen des MHLS veröffentlicht, die in einem systematischen Review mit Fokus auf die psychometrischen Eigenschaften diskutiert werden 12 . Darin noch nicht enthalten ist die Ende 2024 veröffentlichte deutsche Version des MHLS -GER von Fischer et al. 13 , die für ihre Skala 4 Faktoren (Wissen, Informationssuche, Stigmatisierung, Soziale Distanz) vorschlagen. Bei anderen Übersetzungen wurden Items aus psychometrischen oder anderen Gründen entfernt (vgl. 12 ) und auch unterschiedlich viele Faktoren extrahiert. Insgesamt ist das Konzept der MHL noch nicht abschließend festgelegt. Die unterschiedlichen Faktorlösungen weisen auf die möglicherweise lückenhafte Abdeckung des MHL Konzepts hin. Es fehlen Vorschläge für das Scoring (Item-Antworten unterschiedlich reliabler Subskalen werden gleich gewichtet zu einem Gesamtscore summiert) und eine kulturübergreifende Validierung steht noch aus.

Das Ziel der vorliegenden Arbeit war die Erstellung und Überprüfung einer für DSM-5 aktualisierten, verkürzten und für Deutschland angepassten Version. Es wurden psychometrische Eigenschaften, die faktorielle Struktur und die Konstruktvalidität durch Vergleich einer 4- und 6-Faktorenlösung überprüft. Da das Konstrukt der MHL noch in Entwicklung ist, wurden Variablen, die mit der Stigmatisierung psychischer Erkrankungen verbunden sind, für einen Abgleich mit MHL aufgenommen. Das Projekt (AZ 168/22: Wissenschaft im Trialog: Scham & Stigmatisierung im Kontext psychiatrischer Erkrankungen) wurde von der zuständigen lokalen Ethikkommission des FB-Medizin der JLU mit einem positiven Votum ausgestattet.

## Methoden

### Mental Health Literacy Scale (MHLS)

Der Fragebogen wurde in einem mehrstufigen Übersetzungsprozess in enger Anlehnung an die Empfehlungen der COSMIN Study Design Checklist, Paragraph „Translation Process“, (2019, Seite 29; 14 ) aus dem Englischen ins Deutsche übertragen. Die Schritte umfassten mehrfache Übersetzung und Rückübersetzung unabhängig durch jeweils 3 muttersprachliche Übersetzerinnen, davon eine mit fachlicher Expertise, abschließend ein Experten-Review.

### Modifikationen

Die a priori Modifikationen der MHLS-DE betrafen nach Rücksprache mit dem Autor des originalen MHLS, Matt O’Connor, Aktualisierungen entsprechend DSM-5. Dazu wurde das Item 5 zur Bedeutung des Begriffs „Dysthymie“ präzisiert und in Item 8 wird nun der Begriff „Substanzgebrauchsstörung“ benutzt. Die Items 14 und 15 zur ärztlichen Schweigepflicht wurden an die deutsche Rechtslage angepasst und angesichts der komplexen Ausnahmeregelung des rechtfertigenden Notstands (§ 34 StGB) vereinfacht. Dazu wurden beide Items zu einer Frage, ob die Schweigepflicht auch gegenüber Familienangehörigen gilt, zusammengefasst. Auf der Basis der Empfehlungen aus der französischen Übersetzung 15 wurde der ursprünglich 35 Items umfassende Fragebogen auf 26 Items gekürzt – das sind neun weniger als das australische Original und fünf weniger als die kürzlich veröffentlichte MHLS-GER 13 .

### Psychologische Variablen

Variablen, die potentiell einen Effekt auf die Psychische Gesundheitskompetenz aufweisen könnten, wurden in die Datenerhebung eingeschlossen. Das waren in Anlehnung an das Stressverarbeitungsmodell für Stigma bei psychischen Erkrankungen nach Rüsch et al. 16 , der Fragebogen zu Stigma-Stress 17 , der Fragebogen Stigmatisierung (Stig9) 18 ; die Skala zur Allgemeinen Selbstwirksamkeitserwartung (SWE) 19 , der WHO-Fragebogen zum Wohlbefinden (WHO-5) 20 21 und der *Test of Self-Conscious Affects* (TOSCA) zur Erfassung des Schamgefühls 22 . Ergänzend wurden „Psychische Symptomlast“ der American Psychiatric Association 23 und „Psychologische Flexibilität“ mit dem Fragebogen zu Akzeptanz und Handeln (FAH-II) 24 sowie demographische Variablen einschließlich des Vorliegens einer diagnostizierten psychischen Erkrankung (Selbstauskunft) erfragt.

### Stichprobe

Es wurde eine Online-Datenerhebung durchgeführt. Die Einladung zur Teilnahme an der Studie erfolgte via Soziale Medien, Aushängen mit QR-Code und E-Mail-Verteiler. Das daraus resultierende Untersuchungskollektiv lässt sich als Community-Stichprobe beschreiben, welche nicht repräsentativ (für die deutsche Gesamtbevölkerung), aber für post-ex-facto Analysen geeignet ist. Die Studienteilnahme erfolgte vollständig anonym. Die teilnehmenden Personen wurden darüber und über die Verwendung der Daten aufgeklärt. Sie konnten jederzeit die Teilnahme beenden und ihre bis zu diesem Zeitpunkt abgegebenen Daten vollständig löschen. Eine mehrfache Absendung wurde durch die Verwendung von Cookies eingeschränkt. Der so erhaltene Datensatz wurde bezüglich Qualitätskriterien für Online-Erhebungen überprüft 25 . Die Abschätzung der mindesten benötigten Stichprobengröße wurde mit G*Power 26 auf N≥178 geschätzt (multiple lineare Regression, ρ ^2^ =0,09, α=0,05, Power=0,95)

### Statistische Analyse

Reliabilität wurde entsprechend der Klassischen Testtheorie (CTT) als interne Konsistenz mit McDonald‘s ω berechnet, das anders als Cronbach‘s α nicht voraussetzt, dass alle Items gleich stark mit der latenten Variable korreliert sind, was in empirischen Datensätzen zumeist der Fall sein wird. Cronbach’s α wurde berechnet um eine Vergleichbarkeit mit anderen Übersetzungen der MHLS in der Literatur zu ermöglichen. Für beide Reliabilitätsmaße wird r>0,7 als minimale Ausprägung akzeptiert.

Die faktorielle Struktur des Fragebogens wurde mit einer explorativen Faktoranalyse untersucht (Maximum Likelihood Extraktion; Oblimine Rotation der Faktoren). Die Konstruktvalidität wurde mit einer konfirmatorischen Faktoranalyse (CFA) überprüft. Es wurde die 4-Faktoren-Lösung und die 6-Faktoren-Lösung basierend auf dem MHLS und dem MHLS-FR verglichen 15 . Die Voraussetzungen für Faktoranalysen wurden mit dem Bartlett’s Test auf Sphärizität, und dem Kaiser-Meyer-Olkin Kriterium für die Angemessenheit der Probennahme (KMO) geprüft. Die Güte der Modellanpassung wurde mit den verbreiteten Modell-Fit-Indizes Chi-Quadrat-Test (χ2<0,001), Tucker-Lewis Index (TLI≥0,95), Comparative Fit Index (CFI≥0,95), Root Mean Square Error of Approximation (RMSEA≤0,06) und dem Standardized Root Mean Square Residual (SRMR≤0,08 ) überprüft. In Klammern stehen die üblicherweise empfohlenen und hier angewandten kritischen Werte. Es wurden Modifikationsindizes für Faktorladungen und die Kovarianz berechnet, um mögliche Schwachstellen des Modells zu identifizieren bzw. zu re-modellieren.

Ein Gesamtmodell für die Vorhersage der MHLS-DE-Faktoren aus bzw. die Mediation durch die erhobenen psychologischen Variablen und Gruppeneffekte wurden in einem Pfadmodell untersucht. Die Güte der Modellanpassung wurde wie für die CFA beschrieben geprüft.

Alle Analysen wurden mit jamovi 27 durchgeführt. Es wurden dabei die R-packages *psych* 28 und *lavaan* 29 benutzt.

## Ergebnisse

### Stichprobe

An der Online-Erhebung nahmen 533 Personen teil, in 53 Fällen war die Umfrage begonnen, aber nicht zu Ende geführt worden, sodass 480 Fälle vollständige Fälle verblieben. Die Stichprobenbeschreibung ist in Tab. 1 zusammengefasst. Es nahmen mehr Frauen in der 3. Lebensdekade mit mehr als 12 Bildungsjahren teil. Die teilnehmenden Personen waren überwiegend muttersprachlich deutsch, vorwiegend in einem angestellten Beschäftigungsverhältnis, und nicht Teil in einer organisierten Gemeinschaft (Verein, Kirche, etc.); 38,5% gaben an, eine diagnostizierte psychische Erkrankung zu haben.

**Table:** **Tab. 1** Stichprobencharakteristik

Stichprobencharakteristik											
N=480, Fehlend=53											
Alter (Dekade)	n	%	Geschlecht (sozial)	n	%	Muttersprache	n	%	Wohnsituation	n	%
18–20	32	6,7	weiblich	380	79,2	deutsch	451	94,0	allein in Haushalt	129	26,9
21–30	264	55,0	männlich	76	15,8	andere	29	6,0	mit Familie, in Wohngruppe	175	36,5
31–40	66	13,8	beides	9	1,9				mit Partner in Haushalt	164	34,2
41–50	36	7,5	weder noch	12	2,5				in Beziehung, eigener Haushalt	12	2,5
51–60	54	11,2	anderes	3	0,6						
61–70	13	2,7									
>70	15	3,1									
**Schulbildung**			**Berufstätigkeit**			**Aktivität (Verein, Kirche, …)**			**Erkrankung**		
<10	12	2,5	angestellt	330	68,8	ja	170	35,4	nein	295	61,5
10	21	4,4	selbständig	20	4,2	nein	310	64,6	ja	185	38,5
12	60	12,5	ohne Erwerbsarbeit	130	27,1						
>12	387	80,6									

Anmerkung: n … absolute Häufigkeiten,% relative Häufigkeiten bezogen auf N=480

### Reliabilität

Die Reliabilität wurde als interne Konsistenz (Cronbach’s **α** , McDonalds **ω** ) bestimmt. In Tab. 2 sind die Schätzungen der Reliabilität für jede Subskala bzw. für jeden Faktor der CFA dargestellt. Für den Vergleich mit den bereits veröffentlichten Übersetzungen wurde **α** auch für den gesamten Fragebogen geschätzt, obwohl das wegen der unterschiedlichen Anzahl an Antwortkategorien für die Items 1–11 (4 Kategorien) und 12–26 (5 Kategorien) und der bisher nicht bestätigten Eindimensionalität des Konstrukts womöglich nicht voll korrekt ist. Die Reliabilitätsschätzungen für die Faktoren weisen diese Probleme nicht auf, dafür aber eine unterschiedliche Anzahl von Items, welche die Höhe der Reliabilitätsschätzung beeinflusst.

**Table:** **Tab. 2** Ergebnisse der konfirmatorischen Faktorenanalysen für die 4-Faktor und die 6-Faktor Lösungen, Es ist die interne Konsistenz für jeden Faktor und die gesamte Skala als Cronbach’s **α** und McDonald’s ω angegeben, Im unteren Teil der Tabelle sind die korrespondierenden Schätzer der Modellgüte, CFI, TLI, SNMR, RMSEA, AIC, BIC, und χ2 dargestellt,




### Faktorielle Struktur

Eine explorative Faktoranalyse mit Maximum-Likelihood Extraktion und obliminer Rotation ermittelt auf der Basis der Parallelanalyse 4 Faktoren. Geordnet nach der Größe der Faktorenladungen umfasst Faktor 1 die Items 20–26, der Faktor 2 die Items 12–15, Faktor 3 die Items 1–7 und der Faktor 4 die Items 16–19. Die Items 8, 9 und 11 werden auf der Basis von Faktorladungen>0,3 keinem Faktor zugeordnet, haben jedoch eine hohe Eigenvarianz (MHLS-DE-08: 1-h ^2^ =0,96; MHLS-DE-09 1-h ^2^ =0,97; MHLS-DE-11 1-h ^2^ =0,84). Die Reliabilitätsschätzung für die Skala ändert sich durch Entfernung dieser Items nicht (McDonalds _ohne8,9,11_ **ω** =0,86). Die Güte der Modellpassung ist zufriedenstellend (RMSEA=0,048±0,007; TLI=0,909; χ²=416.736, df=227, p<0,001).

### Konstruktvalidität

Die Voraussetzungsprüfung ergab keinen Hinweis, dass eine CFA nicht durchgeführt werden sollte. Der Bartlett Test auf Sphärizität ist mit χ²=3338,853 (df=325; p<0,001) signifikant, d. h. die Korrelationsmatrix entspricht nicht der Einheitsmatrix. KMO=0,858, die einzelnen MSA (Measure of Sampling Adequacy) sind alle größer als MSA=0,05. Den kleinsten Wert zeigt MHLS-DE-09 mit MSA=0,541 die MSAs für die anderen Items liegen alle über ,7 (n=9), über ,8 (n=13) und über ,9 (n=4). Tab. 2 zeigt die Zusammenfassung der Ergebnisse der CFA für die 4 Faktoren des MHLS-GER und für die 6 Faktoren der MHLS-FR. Alle Maße für die Modellgüte zeigen einen besseren Fit des 6-Faktormodells, das auch das Problem des Item MHLS-DE-09 in der 4-Faktoren-Lösung behebt, in dem dieses Item gemeinsam mit MHLS-DE-08 einen Faktor bildet. Durch Berücksichtigung der durch Modifikationsindikatoren angezeigten Kovarianzen von MHLS-DE-01 und MHLS-DE-02, sowie MHLS-DE-25 und MHLS-DE-26 verbesserte sich der Modellfit so, dass nun alle Modell-Fitindices die geforderten Grenzwerte klar übertreffen.

### Effekte von anderen psychologischen Variablen auf die MHL

Die Vorhersage von Psychischer Gesundheitskompetenz umfasste die Prädiktoren Stigmatisierung (Stig9), Selbstwirksamkeitserwartung (SWE), Psychische Flexibilität (FAH-II) und als Mediatoren Stigma-Stress und Schamempfinden (TOSCA).

Die Modellgüte wurde mit dem SEMj Modul in jamovi 30 untersucht und mit CFI=0,994, SRMR=0,019, RMSEA=0,034, Globaler Modellfit Χ ^2=^ 5,397, df=4, p=0,249 als voll gegeben angenommen.

**Abb. 1:**
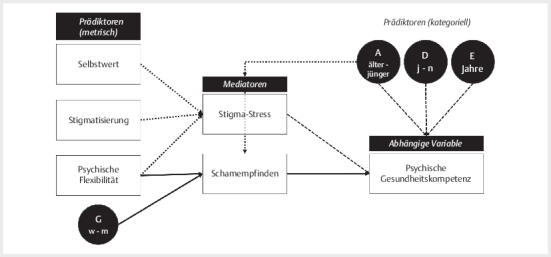
Konzeptionelles Pfadmodell der Mediationsanalyse mit den metrischen Prädiktoren Selbstwirksamkeitswerwartung (SWE), Stigmatisierung (Stig9), Schamerleben (TOSCA) und den kategoriellen Prädiktoren Soziales Geschlecht (f, m), Alter ([18–40 : jünger], [>40 : älter]), Schulbildung ([<10, 10, 12,>12 Jahre]) und Psychische Erkrankung (ja vs. nein). Als Mediatoren auf die abhängige Variable Psychische Gesundheitskompetenz (PGK) wurden Stigma-Stress und Schamerleben (TOSCA) geprüft. Pfeile repräsentieren Pfade mit z>2. Durchgezogene Pfeile stellen die Mediationseffekte [Psychische Flexibilität ⇒ Schamempfinden ⇒ Psychische Gesundheitskompetenz] und [Geschlecht ⇒ Schamempfinden ⇒ Psychische Gesundheitskompetenz] dar. Die Statistiken sind in Tab. 3 aufgeführt.

Die Mediationsanalyse wurde mit dem jamovi-Modul GAMLj 31 unter Hinzunahme der dichotomen Variablen (Geschlecht, Erkrankung) und der ordinalen Variablen (Schulbildung in Jahren und Alter) durchgeführt. Die Unterschiede für diese Variablen wurden durch Kontrastbildung für Geschlecht [weiblich -männlich], für Erkrankung [ja-nein], für Schulbildung in Jahren [10 -<10] [12 -<10] [>12 -<10] und für Alter [18–40 : jünger], [>40 : älter], im Modell repräsentiert. Regressionspfade mit z>2 werden als bedeutsam gewertet. Die Ergebnisse sind in Tab. 3 dargestellt, das Pfadmodell mit signifikanten Verbindungen ist Abb. 1 graphisch repräsentiert.

**Table:** **Tab. 3** Ergebnisse der Mediationsanalyse mit den metrischen Prädiktoren Selbstwirksamkeit (SWE), Stigmatisierung (Stig9), Schamerleben (TOSCA) und den kategoriellen Prädiktoren Soziales Geschlecht (f, m), Alter ([18–40 : jünger], [>40: älter]), Schulbildung ([<10, 10, 12,>12 Jahre]) und Psychische Erkrankung (ja vs, nein), Als Mediatoren auf die abhängige Variable Psychische Gesundheitskompetenz (PGK) wurden Stigma-Stress und Schamerleben (TOSCA) geprüft, Es werden die Ergebnisse für direkte und indirekte Pfade dargestellt,

			95% CI				
Effekt für Pfad	Est.	SE	Lower	Upper	β	z	p
***Indirekter Pfad***							
Stigmatisierung ⇒ TOSCA_Score ⇒ PGK	0,105	0,133	−0,156	0,366	0,007	0,790	0,430
Stigmatisierung ⇒ Stress_Score ⇒ PGK	−0,288	0,166	−0,612	0,037	−0,019	−1,734	0,083
Selbstwert ⇒ TOSCA_Score ⇒ PGK	−0,283	0,221	−0,717	0,151	−0,016	−1,279	0,201
Selbstwert ⇒ Stress_Score ⇒ PGK	0,688	0,354	−0,006	1,382	0,039	1,944	0,052
**Psych, Flexibilität ⇒ TOSCA_Score ⇒ PGK**	**0,520**	**0,202**	**0,124**	**0,915**	**0,083**	**2,576**	**0,010**
Psych, Flexibilität ⇒ Stress_Score ⇒ PGK	−0,158	0,096	−0,346	0,029	−0,025	−1,658	0,097
Psych, Erkrankung1 ⇒ TOSCA_Score ⇒ PGK	0,026	0,182	−0,331	0,383	0,001	0,142	0,887
Psych, Erkrankung1 ⇒ Stress_Score ⇒ PGK	−0,065	0,143	−0,345	0,215	−0,003	−0,455	0,649
**Soz, Geschlecht ⇒ TOSCA_Score ⇒ PGK**	**0,664**	**0,309**	**0,059**	**1,269**	**0,027**	**2,151**	**0,031**
Soz, Geschlecht ⇒ Stress_Score ⇒ PGK	−0,178	0,173	−0,517	0,161	−0,007	−1,031	0,302
Schulbildung1 ⇒ TOSCA_Score ⇒ PGK	0,499	0,751	−0,974	1,971	0,010	0,664	0,507
Schulbildung1 ⇒ Stress_Score ⇒ PGK	−0,452	0,598	−1,625	0,720	−0,009	−0,756	0,450
Schulbildung2 ⇒ TOSCA_Score ⇒ PGK	0,704	0,680	−0,630	2,038	0,026	1,035	0,301
Schulbildung2 ⇒ Stress_Score ⇒ PGK	0,512	0,541	−0,547	1,572	0,019	0,948	0,343
Schulbildung3 ⇒ TOSCA_Score ⇒ PGK	0,554	0,638	−0,695	1,804	0,023	0,869	0,385
Schulbildung3 ⇒ Stress_Score ⇒ PGK	0,242	0,479	−0,696	1,180	0,010	0,505	0,614
Alter ⇒ TOSCA_Score ⇒ PGK	−0,392	0,230	−0,842	0,058	−0,019	−1,707	0,088
Alter ⇒ Stress_Score ⇒ PGK	−0,336	0,209	−0,746	0,075	−0,016	−1,602	0,109
***Direkter Pfad***							
Stigmatisierung ⇒ PGK	0,860	0,813	−0,733	2,454	0,056	1,058	0,290
Selbstwert ⇒ PGK	1,820	1,280	−0,690	4,329	0,104	1,421	0,155
Psych. Flexibilität ⇒ PGK	−0,193	0,536	−1,244	0,858	−0,031	−0,360	0,719
**Psych. Erkrankung1 ⇒ PGK**	**5,052**	**1,143**	**2,813**	**7,292**	**0,268**	**4,421**	**<0,001**
Soz. Geschlecht ⇒ PGK	1,815	1,266	−0,667	4,297	0,074	1,433	0,152
**Schulbildung1 ⇒ PGK**	**11,022**	**4,586**	**2,032**	**20,011**	**0,228**	**2,403**	**0,016**
**Schulbildung2 ⇒ PGK**	**15,829**	**3,981**	**8,025**	**23,632**	**0,585**	**3,976**	**<0,001**
**Schulbildung3 ⇒ PGK**	**15,375**	**3,810**	**7,908**	**22,842**	**0,650**	**4,036**	**<0,001**
**Alter ⇒ PGK**	**−2,926**	**1,160**	**−5,199**	**−0,652**	**−0,142**	**−2,522**	**0,012**

*Anmerkung.* Betas repräsentieren standardisierte Effektstärken, Zeilen mit z>2 wurden fett gedruckt; CI … Konfidenzintervall

Das Modell mit den Mediatoren Stigma-Stress und Scham zeigt direkte Effekte von Alter, Schulbildung und dem Vorliegen einer psychischen Erkrankung auf die Psychische Gesundheitskompetenz. Personen mit längerer Schulbildung, mit psychischer Erkrankung und niedrigerem Alter zeigten bessere Psychische Gesundheitskompetenz. Höheres Alter ging mit höherem Stigma-Stress und geringerem Schamerleben einher. Das Geschlecht hatte keinen direkten Effekt auf Psychische Gesundheitskompetenz, aber einen Effekt auf Schamerleben, mit einem stärkeren Zusammenhang bei Frauen. Im vorgeschlagenen Modell waren keine direkten Effekte von Selbstwert, Stigmatisierung oder Psychische Flexibilität auf Psychische Gesundheitskompetenz evident. Ein Mediator-Effekte durch Schamerleben für Psychische Flexibilität auf Psychische Gesundheitskompetenz konnte dargestellt werden. Dieser Mediationseffekt war für Frauen stärker als für Männer. Die gesamten Ergebnisse sind in Abb. 1 dargestellt, die Statistiken werden in Tab. 3 gezeigt.

## Diskussion

Die Ergebnisse der Validierungsstudien zur strukturellen Validität der MHLS zeigen große Unterschiede 12 . Die vier Faktor-Lösung (mit niedrigen Kommunalitäten) aus dem originalen MHLS wurde in der kürzlich veröffentlichten deutschen Version der MHLS-GER 13 bestätigt. Bei der französischen Übersetzung 15 wurde eine 6-Faktoren-Lösung, für die MHLS- Südafrika eine 3-Faktoren-Lösungen 32 , und für die MHLS-Iran eine 5-Faktoren-Lösung 33 34 beschrieben. Da die Skalen reliabel sind, ist das vorerst kein Mangel, sondern weist darauf hin, dass innerhalb des sehr breit zu fassenden Konstrukts „Psychische Gesundheitskompetenz“ relevante Facetten (methodisch, inhaltlich, kulturell, sprachlich) noch nicht ausreichend erfasst werden 12 35 oder das Konstrukt eben doch mehrdimensional ist.

Trotz konzeptioneller und methodischer Einschränkungen scheint die Erfassung von Psychischer Gesundheitskompetenz in der gegenwärtigen Konzeption gemessen an der Anzahl an Publikationen (2024: 767, https://pubmed.ncbi.nlm.nih.gov/?term=Mental+health+literacy +) international nachgefragt zu sein. In der vorliegenden Studie wird eine überprüfte deutschsprachige Übersetzung der MHLS, die MHLS-DE vorgeschlagen. Etwas zeitgleich wurde bereits die deutsche Übersetzung MHLS-GER veröffentlicht 13 , jedoch mit einem Fokus auf Herzinfarkt-Patienten. Der Vorteil dieser Koinzidenz liegt in einer unmittelbaren Vergleichbarkeit.

Während die MHLS-GER Version die 35 Items der Originalversion beibehielt, orientierte sich die MHLS-DE Version an der auf der Grundlage hoch korrelierender Items auf 26 Items reduzierten französischen Version. Die Faktoren der MHLS-FR wurden mit „Fähigkeit, Störungen zu erkennen“, „Kenntnis von Geschlecht als Risikofaktor“, „Wissen/Einstellung zu Selbsthilfe-Interventionen“, „Wissen über Informationssuche“ und „Stigmatisierung“, jene 4 Faktoren der MHLS-GER mit „Wissen“, „Informationssuche“, „Stigmatisierung“ und „Soziale Distanzierung“ beschrieben.

Die 4 extrahierten Faktoren der MHLS-DE wurden in Anlehnung daran, nach Ladungsgröße absteigend, als „Soziale Ausgrenzung“, „Informationssuche“, und „Wissen und Stigmatisierung“ benannt. Die Ladungen (0,34–0,88) und damit auch die Kommunalitäten sind somit größer als bei O’Connor & Casey 11 , aber etwa vergleichbar mit der MHLS-GER 13 . Die Varianzaufklärung der 4 Faktoren beträgt 39,9%. Drei Items, Items 8, 9 und 11 hatten eine hohe Eigenvarianz, konnten weder einem Faktor zugeordnet werden, noch bilden sie einen gemeinsamen Faktor. Ein Vergleich der strukturellen Validität für die 4- und die 6-Faktoren-Lösung zeigt gemessen an Modellfitparametern eine bessere Passung der 6-Faktoren-Lösung. Dies ist vor allem auf die bessere Einbindung der problematischen Items mit hoher Eigenvarianz zurückzuführen. Für die Reliabilität der Skala spielen diese keine Rolle. Allerdings fällt für die Faktoren 2 und 3, Wissen zu Risikofaktoren und professioneller Hilfe, die interne Konsistenz dann deutlich unter die Akzeptanzgrenze. Eine detaillierte Item- und Skalenanalyse, wie sie mit Modellen der Item Response Theorie (IRT) möglich ist, könnte die Frage nach der Dimensionalität, Itemeigenschaften und Modellpassung voranbringen. Für die persische Übersetzung der MHLS 33 wurde z. B. mit IRT eine unterschiedliche Diskriminationsfähigkeit der Items gezeigt.

Die Vorhersagbarkeit von Psychischer Gesundheitskompetenz wurde in einem Regressionsmodell mit den Prädiktoren Stigmatisierung, Selbstwirksamkeitserwartung, Psychische Flexibilität, Geschlecht, Erkrankung, Schulbildung, Alter und als Mediatoren Stigma-Stress und Schamempfinden überprüft. Das wichtigste Ergebnis zeigte einen Mediator-Effekt des Schamempfindens auf den Zusammenhang von psychischer Flexibilität mit psychischer Gesundheitskompetenz, insbesondere bei Frauen. Schamerleben, das mit sozial zugeschriebenen Geschlechterrollen assoziiert ist 36 , scheint demnach besonders für Frauen ein wichtiger Einflussfaktor in der Auseinandersetzung mit psychischer Gesundheit zu sein.

Die Einschränkungen der Arbeit ergeben sich aus den oben diskutierten Problemen mit der Fundierung des Konstrukts Psychische Gesundheitskompetenz und den Einschränkungen durch die Konstruktion des immerhin etablierten Fragebogens. Dennoch ermöglicht die gute Reliabilität der Skala die zuverlässige Erfassung von MHL entsprechend ihrer theoretischen Konzeption. Eine weitere Einschränkung ist bedingt durch einen Alters-, Geschlechts- und Bildungs-Bias der Stichprobe, jedoch mit einem hohen Anteil (≈40%) von Personen, die angeben, psychisch erkrankt zu sein. Die für die Beurteilung der Testgüte zentrale Frage, welche Population in einer MHLS-Studie repräsentiert werden soll, ist nach dem Wissenstand der Autoren bislang noch nicht systematisch diskutiert worden.

### Konsequenzen für Klinik und Praxis

Mit der MHLS-DE liegt eine alternative deutschsprachige Version des MHLS vor. Mit 26 Fragen ist er kürzer aber gleich reliabel wie der originale Fragebogen.

Vor allem Frauen mit stärkerem Schamerleben und der Fähigkeit, trotz schmerzlicher Erfahrungen das eigene Leben wertzuschätzen und Sorgen nicht nur als Bedrohung zu erleben, sondern flexibel handeln zu können 24 entwickeln eine bessere Psychische Gesundheitskompetenz.

Dieses Ergebnis legt die Verwendung akzeptanzbasierte Strategien zur Verbesserung des Gesundheitsverhaltens nahe.

## Fördermittel

Gießener Aktionsbündnis für Seelische Gesundheit e.V. — Stipendium/Pia Höhler Hessisches Ministerium für Wissenschaft und Kunst — http://dx.doi.org/10.13039/501100003495; LOEWE 1/16/519/03/09.001 (0009)/98 (Christoph Mule
